# Factors associated with sanitary conditions of food and drinking establishments in Addis Ababa, Ethiopia: cross-sectional study

**DOI:** 10.11604/pamj.2017.28.237.13734

**Published:** 2017-11-15

**Authors:** Eyerusalem Kassa Mendedo, Yemaneh Berhane, Biniyam Tadesse Haile

**Affiliations:** 1 Addis Ababa City Administration Health Bureau, Arada Sub-city Health Office, Addis Ababa, Ethiopia; 2 Addis Continental, Institute of Public Health, Addis Ababa, Ethiopia; 3 Jimma University, Institute of Health, Jimma, Ethiopia

**Keywords:** Food and drinking establishments, sanitary conditions, Arada sub-city

## Abstract

**Introduction:**

Food borne illness has been a global challenge and it persisted as a major public health problem, which consumes significant amounts of health care resources, particularly in the developing world. Poor sanitary conditions of food and drinking establishments are the major cause for the occurrence of food borne illness. This study assessed sanitary conditions of food and drinking establishments in Arada sub-city, Addis Ababa, Ethiopia.

**Methods:**

A cross-sectional study design with stratified simple random sampling technique was used. Data were collected from 587 licensed food and drinking establishments and their managers, using interviewer administered questionnaire and observation checklist. The data were entered using Epi info version 3.5.3 and analyzed using SPSS version 20. Binary and Multi-variable logistic regression analyses were conducted.

**Results:**

The study showed 58.8% of food and drinking establishments were under poor sanitary conditions; only 16.5% of the establishments had a proper liquid waste disposal facility, and only 7.2% had a suitable dish washing facility. Availability of trained managers on hygiene and sanitation (AOR = 2.56, 95% CI: 1.66-3.94); inspection from the respective body (AOR = 4.41, 95% CI: 2.9-6.8) and the distance between kitchen and toilet (AOR = 1.8, 95% CI: 1.1-3.0) were associated factors which affected sanitary conditions.

**Conclusion:**

A majority of the establishments had poor sanitary conditions; where an absence of sanitary facilities for waste management was major cause. Regulatory bodies should conduct regular inspection on the establishments to promote and ensure proper hygiene and sanitation practices.

## Introduction

Food borne diseases have been an issue for all societies [1]. Increases in the incidence of food borne diseases, often associated with outbreaks, which end up with threatening global public health security [2,3]. The risk of epidemics has been observed in the era of globalization that is characterized by increased frequency of travels and eating outside of the home [4,5]. According to World Health Organization (WHO) estimates, 2 million people in developing countries die due to food borne diseases each year; whereas, 30% of people in developed countries have a medical condition from food borne diseases each year [6]. Like other developing countries, Ethiopia is affected by the increasing burden of food-borne diseases; major food safety concerns are caused by physical, chemical, and microbiological contaminants. A summary report on out-patient visits of the Ministry of Health, released in 2014, indicates annual incidence of food-borne illnesses ranged from 3.4% to 9.3%, the median being 5.8% [7]. Food borne diseases can be caused by different pathogenic organisms (e.g. bacteria or viruses) that have contaminated them at some part of the food chain, between farm and fork [8]. Chemical contamination of foodstuffs, including methyl mercury, lead, arsenic, dioxins and aflatoxins (among others) may cause acute and chronic health effects such as neuro-developmental disorders, cardiovascular disease, cancers and renal disease [6,8]. Despite the considerable burden of food borne diseases on health and socio- economic development, food safety interventions have remained at the least priority in developing regions [1]. According to various studies, poor knowledge and practice of hygiene and sanitation, lack of basic sanitary facilities/infrastructures in food service establishments, and negligence in safe food handling are major causes of poor sanitary conditions of food and drinking establishments [3, 9-12]. Hence, the current study is aimed to gain understanding of factors affecting sanitary conditions of food and drinking establishments. It will provide systematic information for policy makers and planners in designing interventions to improve the sanitary conditions of food and drinking establishments.

## Methods

**Study setting, and population:** The study was conducted in Arada Sub-city, which is one of the 10 sub-city administrations in Addis Ababa. It has a total of 61,818 households inhabited by a population of 240,165. According to data obtained from the sub-city administration, there are 2,066 licensed food and drink establishments distributed in the 10 districts of Arada sub-city administration. These establishments include: Cafeterias, Hotels, Bars and Restaurants, Juice houses, and Pastries/Bakeries. All the food and drinking establishments, and their respective managers or owners were the source population from which the sample study subjects were selected.

**Study design and sampling**: A cross-sectional survey design was applied to assess sanitary conditions of licensed food and drinking establishments and associated factors. The sample size was determined using single population proportion formula for finite population with 95% confidence level, 78.7% prevalence of poor sanitary condition in the study area [10] and 4% margin of error. Adding 10% non-response rate, the final sample became 587. The study used stratified simple random sampling techniques; list of all establishments in the study area was used as sampling frame and to stratify establishments by type (i.e. hotels, bar and restaurants, cafeterias, juice houses and bakery/pastry). The total sample size was proportionally allocated for each stratum; and study participants were selected using simple random sampling technique.

**Operational definitions of outcome variables**: Outcome variable, which is overall sanitary conditions, was computed by taking summation of eighteen criteria presented in table 3. Each criterion was given a value of 1 for the presence of sanitary condition/facility and 0 for the absence. The sum of these conditions was computed and the mean score of all observations was used as a cut-off point to categorize establishments. Food and drinking establishments with higher than mean value were categorized under **good** sanitary conditions; whereas, whose score was < 11.2 were considered as **poor** sanitary conditions.

**Measurement tools, data collection and data analysis**: Data were collected by environmental health professionals, using a structured pre-tested questionnaire and observation checklist adopted from related literatures. The observation checklist was designed to assess availability, utilization, cleanliness and maintenance status of sanitary facilities that are used for food preparation, cleaning, waste handling and disposal. The raw data was edited, cleaned and entered using Epi-Info version 3.5.3, then exported to SPSS version 20. Data was cleaned in SPSS by running frequencies and cross tabulations. Preliminary frequencies were run to identify missing variables. Binary logistic regression analysis was done to see the association between the dependent and independent variables. Variables with p-value less than 0.25 were taken as candidates of multivariable logistic regression analysis, and Multi variable logistic regression was performed to see the relative effect of independent variables on the outcome. The binary and multivariable analysis results were presented using Odds ratio with 95% confidence interval, and P-value < 0.05 was considered statistically significant.

**Ethical considerations**: The study was approved by Addis Continental Institute of Public health and Haramaya University ethical review committee. Verbal consent was also obtained from each respondent after explaining the purpose of the study. Confidentiality and anonymity was maintained by avoiding personal identifiers.

## Results

**Characteristics of study participants :** The study assessed a total of 587 licensed food and drinking establishments, which consists of: 217(37%) cafeteria, 187(31.9%) bar and restaurants, 23(3.9%) juice houses, 6(14.6%) hotels and 74(12.6%) pastries. From the total 587 participants representing those establishments, 380(64.7%) were male; and mean age of respondents was 36 with standard deviation of ± 11.351 years (Table 1, Table 2). Only 242(41.2%) establishments had good sanitary condition. Among all the establishments 467(85.8%) had functional latrine; while, 386(71.0%) of the latrines were managed properly. The result also showed absence premises or materials for proper solid and liquid waste management (Table 3).

**Table 1:** Characteristics of food and drink establishments by type of service provided, Addis Ababa, Ethiopia, April 2016 (n = 587)

Characteristics (n=587)	Food and Drink Establishment by Type					
	Cafeteria	Bar and restaurant	Juice Houses	Hotels	Pastry (Bakery)	Total Number
**Type of service provided**						
Only food	0	0	23	0	0	23
Food and drinks	217	187	0	0	74	478
Food, drinks and bed	0	0	0	86	0	86
**Ownership of Establishment building**						
Rented from Government	105	81	8	29	26	249
Privately owned	54	39	2	24	18	137
Ranted from Individuals	58	67	13	33	30	201
**Ownership of the establishment service**						
Owned by Individuals	201	178	21	74	72	546
Private Limited Company	12	8	0	11	1	32
Government owned	3	1	2	1	1	8
**Manager of the establishment**						
Owner him/her self	95	81	11	35	29	251
Relative	65	51	10	24	28	178
Hired person	51	53	2	27	17	150
Selected from the public	6	2	0	0	0	8

**Table 2:** Socio demographic characteristics of owners/managers in food and drink Establishments in Arada Sub City, Addis Ababa, Ethiopia, April 2016 (n = 587)

Socio demographic profile of respondents (Managers/Owners)	Total Number	%
**Sex**		
Male	380	64.74
Female	207	35.26
**Educational status**		
No formal Education	23	3.9
Grade 1-6	85	14.5
Grade 7-12	243	41.4
>12 grade	236	40.2
**Age of the Manager in Years**		
18-24	49	8.3
25-34	234	39.9
35-44	193	32.9
45-54	61	10.4
>55 Years	50	8.5

**Table 3:** Sanitary condition of food and drink establishments in Arada Sub City, Addis Ababa, Ethiopia, April 2016 (n = 587)

Criteria for Sanitary Condition	Yes, Frequency	%
Provision of functional hand washing facility	452	83.5
Availability of a functional latrine	467	85.8
Properly managed latrine facility	386	71.0
Availability of container for solid waste storage	267	46.8
Proper liquid waste disposal	89	16.5
Availability of three compartments for dish/glass washing	42	7.2
Availability of store room for non-perishable foods	347	59.1
Availability of separate kitchen room	568	96.8
Availability of functional refrigerator	483	82.3
Availability of piped water supply	500	85.2
Final disposal of solid waste	547	96
Practicability of proper storage of food utensils	419	71.4
Proper drinking water storage materials	178	30.3
Food handlers wearing appropriate outer garment	396	67.5
Food handlers wearing appropriate hair cover	315	53.7
Food handlers health examination card availability in 6 months	337	57.4
Availability of separate dressing room for food handlers	201	35.4
Insect or rodent infestations not found	223	39.3

**Factors associated with sanitary conditions of establishments:** The multi variable logistic regression analyses result shows that age of the manager, managers' training on food hygiene, inspection by regulatory body, construction of establishments building using brick, distance between kitchen and toilet and use of fire wood for cooking were significantly associated with sanitary condition of food and drinking establishments, with p < 0.05 at 95% CI. Accordingly, food and drinking establishments which are managed by individuals who received training on food hygiene where 2.56 times more likely to have good sanitary condition compared to their counter parts (AOR = 2.56, at 95% CI: 1.7-3.9). On the other hand, establishments which received at least one inspection visit in the past six months were 4.4 times more likely to be in good sanitary condition compared to those were not visited within the specified period (AOR = 4.4, at 95% CI: 2.9-6.8). The study also showed establishments with more than 6 meters distance between their kitchen and toilet had better sanitary condition, as compared to those with 6 or less meter distance (AOR = 1.8, at 95% CI: 1.1-3.0). Moreover, establishments which don't use firewood as major source of energy for cocking had better sanitary condition relative to those using firewood (AOR= 2.0, at 95% CI: 1.3-3.0) (Table 4).

**Table 4:** Factors associated with sanitary condition of food and drinking establishments in Arada sub-city, April2016 (n=587)

Characteristics	Overall Sanitary Condition		Un-adjusted OR	(95%CI)	Adjusted OR	(95%CI)
	Good	Poor				
**Age of manager**						
18-24	16	33	1.00	-	1.00	-
25-34	89	145	1.3	(0.7-2.4)	2.1*	(1.0-2.5)
35-44	83	110	1.6	(0.8-3.0)	2.5*	(1.1-5.4)*
45-54	27	34	1.6	(0.7-3.6)	3.2*	(1.2-8.6)*
Above 55	27	23	2.4	(1.1-5.5)	4.1*	(1.4-11.6)*
**Food hygiene training**						
taken by manager						
Yes	123	107	2.3	(1.6-3.2)	2.6*	(1.7-3.9)*
No	119	208	1.00	-	1.00	-
**Establishment inspected**						
in 6 months						
Yes	151	96	4.3	(3.0-6.1)	4.4*	(2.9-6.8)*
No	91	249	1.00	-	1.00	-
**Building constructed of brick**						
Yes	223	259	3.9	(2.3-6.6)	2.3*	(1.1-4.8)*
No	19	86	1.00	-	1.00	-
**Distance between** **kitchen and toilet**
					
<6 meters	176	275	1.00	-	1.00	-
> 6 meters	66	51	2.0	(1.3-3.0)	1.8*	(1.1-3.0)*
**Firewood used for** **cooking**						
Yes	112	224	1.00	-	1.00	-
No	130	121	2.15	(1.5-3.0)	2.0*	(1.3-3.0)*

Note: **1.00** shows the reference category; whereas,

* shows variables/categories with significant association at P < 0.05.

## Discussion

This study revealed that a greater proportion (58.8%) of food and drinking establishments were found to be in poor sanitary condition; mainly due to inadequate solid and liquid waste management practice, poorly managed toilets, and absence of proper drinking water storage materials. This finding is lower than similar cross-sectionals studies conducted in Bahirdar town (78.7%) and Benin city, Nigerian (69.2%) [4,10]. The discrepancy could be due to difference in their level of urbanization and socio economic characteristics of the study areas. Food and drinking establishments are places where mass food preparation is undertaken, maintaining their sanitary condition ensures production of healthy food. Poor sanitary conditions have causal linkage with incidence of food borne diseases and outbreaks [13]. A vast majority of establishments 92.2% in the study area had toilet facility; this finding is consistent with result of the study done in Bahirdar 93.2%; and lower than findings of similar studies from Mekelle 97.0% [10,14]. On the other hand, out of the establishments having toilet facility, only 71% were properly managed. This finding is in line with a study conducted in Zeway town, in which 75% of establishments had properly managed latrine facilities [15]. Mere availability of toilet facilities does not ensure good sanitary conditions; unsanitary and soiled toilets create favorable breeding environments for insects and rodents which will carry pathogenic micro-organisms and intestinal parasites resulting in the contamination of food and utensils/equipment's; this in turn will result in occurrence of food borne illness [3,14].

Most of the establishments (97.1%) had some kind of refuse receptacles which can be used for solid waste collection and storage. However, only 46.8% of these establishments had proper type of receptacles, recommended by the local regulatory body. In contrast, this finding is higher than a similar study conducted in Bahirdar in which only 33.6% of establishments had proper receptacles for solid waste collection and storage [10]. To avoid unsanitary conditions, refuse receptacles must be constructed and maintained in a manner that will not be damaged due to the moist content of garbage and sharp materials. Moreover, in appropriate receptacles will create favorable conditions for insects such as flies to multiply and contaminate food and utensils. To avoid this, receptacles should be made from durable and heavy-duty materials, easy to clean, handle and transport, and it should have a lid [16]. The current study showed that only 7.2% of the establishments had three compartment dish and glass washing basin. This figure is much lower than findings of similar studies conducted in Mekelle (46%) and Zeway (19%) [14,15]. Inappropriate dishwashing practices contribute to the transmission of various diseases such as TB, influenza, typhoid and other feco-oral diseases. One of the most widely used and accepted methods of food utensil washing method is the three compartment sink or washing basin, which can be used to wash, rinse and sanitize food utensils and equipment's [3,5,8].

The current study revealed that managers who received training on food safety were 2.56 times more likely to maintain hygienic conditions of their establishments compared to their counter parts (AOR = 2.56 at 95% CI: 1.66-3.94). In line with this, a study conducted in Addis Ababa showed that training is significantly associated with improved sanitation (AOR = 1.52 at 95% CI: 1.05-2.90) [17]; moreover, similar study conducted in Ohio showed managers who received training had significantly fewer critical violations than food service facilities managed by untrained personnel [18]. Various studies showed that managers' knowledge and training about hygiene and sanitation have a direct influence on the overall sanitary condition of establishments. They play a vital role by ensuring availability and cleanliness of sanitary facilities, proper waste management and food safety practices [12,19,20]. Managers who received food hygiene and sanitation training were also associated with a reduced risk for food born disease outbreak [21]. This indicates training has an effect on improving the knowledge and practice of mangers in maintaining sanitary condition of their establishments. The study also shows regular inspection visits from regulatory bodies is associated with sanitary outcomes; where, establishments that received at least one inspection visit within the past 6 months were 4.4 times more likely to have good sanitary condition. Similarly, a study done in Mekelle town showed that cleanliness (sanitary condition) is significantly associated with the presence of inspection by regulatory bodies (AOR = 2.13 at 95% CI: 1.20-3.80) [14]. Frequent inspection visits of establishments, supplemented by education is an effective mechanism to improve and maintain sanitary conditions of food and drinking establishments [8,14]. This study has the following strength and weakness: due to its cross-sectional design the study shows sanitary conditions at the instant of data collection, which may vary with time and situations. On the other hand, conducting observation to assess sanitary conditions of the establishments will reduce respondent bias, and it can be considered as strength of the study.

## Conclusion

This study concluded that a majority of the establishments were found to be in poor sanitary condition. A major contributor of poor sanitary conditions include: poor liquid and solid waste management facilities, an absence of proper dish washing facilities, and poor management of toilet facilities. Regular inspections, manager training on of food hygiene, age of managers, the distance between toilet and kitchen, the use of fire wood for cooking, and the use of brick in construction of the establishment are significant factors that determine the sanitary conditions of food and drinking establishments. Since inspection visits are associated with improved sanitary conditions, regulatory bodies should strengthen and improve the frequency of their visits for food and drinking establishments. Concurrently, providing training on hygiene and sanitation for managers of food and drinking establishments will help to improve and/or maintain sanitary conditions. We also suggest further study with mixed method approach to explore the major causes of poor sanitary condition between food and drinking establishments in the area.

### What is known about this topic

- Absence of proper solid waste management system in food and drinking establishments greatly affects the sanitary condition;
- Poor sanitary condition of food and drinking establishment has causal effect on occurrence of food borne diseases and outbreaks;
- Managers' knowledge about hygiene and sanitation has direct influence on overall sanitary condition of establishments.

### What this study adds

- This study revealed majority of food and drinking establishments had poor sanitary conditions; this implies their conditions may lead to or cause occurrence of food borne illness. Food facilities and equipment must be constructed and be maintainable to ensure that they can be effectively and efficiently cleaned and sanitized over their life;
- Absence of trained manager was significant factors associated with poor sanitary conditions of establishments; in contrary, majority of the establishments were managed by untrained managers; this shows importance of training in enhancing adherence of managers to proper hygiene and sanitation standards and practices;
- The study also showed strong association between poor sanitary conditions and absence of inspection visit from regulatory bodies; this shows the need to conduct for frequent evaluation of establishments, to take corrective actions which will in turn improve sanitary conditions.

## Competing interests

The authors declare no competing interest.

## Authors’ contributions

All authors had substantial contributions to conception and design, data analysis and interpretation. Eyerusalem Kassa contributed in the inception, design, data analysis, and manuscript preparation. Professor Yemane Berhane critically edited, reviewed and corrected the article. Biniyam Tadesse reviewed and corrected the article; moreover he contributed during the manuscript preparation. All authors have read and approved the final manuscript.
